# Fifteen years of rifampicin-resistant TB control in Niger: high countrywide treatment uptake and success

**DOI:** 10.5588/ijtldopen.25.0455

**Published:** 2026-01-09

**Authors:** I. Maman Lawan, T. Decroo, M.B. Souleymane, A. Soumana, C.L. Aboubacar, A. Gagara, R. Hamissou Moussa, A.A. Kabirou Amoussa, I. Hamidou, S. Hamidou Moussa, M. Adamou, E. Adehossi, S. Mamadou, B. Catherine de Jong, L. Rigouts, N. Lorent, M. Doutchi, A. Piubello

**Affiliations:** 1 Damien Foundation, Niamey, Niger;; 2 Institute of Tropical Medicine, TB-HIV Unit, Antwerp, Belgium;; 3 Department of Biomedical Sciences, University of Antwerp, Antwerp, Belgium;; 4 National Tuberculosis Programme, Coordination, Niamey, Niger;; 5 Hôpital National Amirou Boubarcar Djallo, Sercive Pneumo-Phtysiologie, Niamey, Niger;; 6 Faculté des Science de la Santé, Université Abdou Moumouni de Niamey, Niamey, Niger;; 7 Centre Hospitalier Régional de Tahoua, CAT, Tahoua, Niger;; 8 Centre Hospitalier Régional de Maradi, CAT, Maradi, Niger;; 9 Faculté des Science de la Santé, Université André Salifou de Zinder, Zinder, Niger;; 10 Hopital National de Zinder, Sercive Pneumo-Phtysiologie, Zinder, Niger;; 11 Ministère de la Santé Publique, Direction Générale de la Santé Publique, Niamey, Niger;; 12 Institute of Tropical Medicine, Unit of Mycobacteriology, Antwerp, Belgium;; 13 Department of Respiratory Diseases, University Hospital Leuven, Leuven, Belgium;; 14 Department of Chronic Diseases, Metabolism and Aging, BREATHE Laboratory, KU Leuven, Leuven, Belgium;; 15 Damien Foundation, Medical Unit, Brussels, Belgium.

**Keywords:** tuberculosis, rifampicin-resistant TB, treatment attrition, treatment outcomes

## Abstract

**BACKGROUND:**

Few rifampicin-resistant TB (RR-TB) studies show pre-treatment outcomes and definitive treatment outcomes (accounting for RR-TB re-treatment). Our Niger countrywide study covered 15 years to show the trend of RR-TB diagnoses, time-to-treatment initiation, and pre-and on-treatment attrition (loss to follow-up or death).

**METHODS:**

Retrospective study including all Niger RR-TB patients diagnosed between 2008 and 2022.

**RESULTS:**

872 RR-TB patients were diagnosed, 725 (83.1%) started treatment, and 32 required retreatment. Between 2008 and 2013 (phenotypic testing), 2014 and 2018 (regional molecular testing), and 2019 and 2022 (decentralised molecular testing), the annual average first RR-TB treatment initiations increased from 22 to 50 and 85, and the median time to first RR-TB treatment reduced from 260 to 17 and 11 days, by period. Pre-treatment attrition reduced from 34.1% to 12.8% and 12.6%. On-treatment attrition increased from 8.8% to 13.5% and 19.8%. Overall, 81.7% (*N* = 725) experienced definitive treatment success. On-treatment attrition was 18.3%, predicted by older age, female gender, low BMI, RR-TB/HIV co-infection, high baseline bacillary load, and treatment initiation between 2019 and 2022.

**CONCLUSION:**

In Niger, over 15 years of RR-TB control, pre-treatment attrition reduced over time, reflecting better access to care. Treatment success was high, exceeding global success. However, increasing on-treatment attrition should be addressed by targeting high-risk groups.

Control of rifampicin-resistant TB (RR-TB), which is resistant to the most potent TB drug, requires high diagnostic and treatment coverage and high treatment success. Globally, about 410,000 people developed RR-TB in 2022, but only 44% were diagnosed.^1^ The introduction of rapid molecular tests, particularly the GeneXpert MTB/RIF assay (Xpert), has revolutionised RR-TB detection. Among those diagnosed, nearly 99% start treatment.^1^ Despite the introduction of new drugs, like bedaquiline (BDQ), and repurposed ones, like linezolid and clofazimine, global RR-TB treatment success remains low, at 50%–65% for cohorts starting between 2008 and 2022.^1^ Treatment regimens have evolved: long regimens lasting 18 months or more have been replaced by shorter, 6- to 9-month regimens,^2^ with a shift from second-line injectable-containing regimens to all-oral ones.^3^

In Niger, the RR-TB control programme began operating in 2008, in collaboration with the Damien Foundation (DF). National guidelines, informed by local evidence, recommend a standardised approach to managing RR-TB. Before 2014, rifampicin resistance was diagnosed using phenotypic methods. In 2014, the first Xpert machines were implemented to diagnose RR-TB rapidly. Between 2014 and 2018, Xpert testing was available in four of the eight regions. Since 2019, it has been decentralised nationwide, with 71 machines now operational across the country. There are currently four RR-TB treatment units, starting with one in 2008, two in 2010, three in 2014, and four since 2019. RR-TB treatment regimens in Niger have been revised over time. Initially, a 12-month second-line injectable-/fluoroquinolone-containing short treatment regimen (SLI/FQ-STR) was used. In January 2011, the SLI/FQ-STR was shortened to 9 months due to a very low relapse rate.^4^ Since 2021, the SLI/FQ-STR has been replaced by a 9-month all-oral regimen (BDQ/FQ-STR) combining BDQ, levofloxacin (a fluoroquinolone), linezolid, and companion drugs. Most RR-TB studies show data on either RR-TB diagnosis or treatment. A comprehensive view is needed to show how effectively an RR-TB programme contributes to RR-TB control. Data on pre-treatment attrition (either loss to follow-up [LTFU] or death) are rarely reported. Similarly, few studies account for definitive outcomes, including retreatment results after relapse or treatment failure, as this requires a long follow-up period.

A 2020 study from Niger by Piubello et al.^5^ showed high definitive treatment success using the RR-TB care cascade, using BDQ-based regimens for patients with failure or relapse after a first RR-TB treatment. We complement this study by covering a larger study period, thus also including data from the recently introduced all-oral regimen. In addition, we present trends in pre-treatment attrition, time-to-treatment (time between the date of sputum sampling and treatment start), and on-treatment attrition. To the best of our knowledge, no previous study has provided a countrywide, longitudinal analysis of RR-TB diagnosis, care, and control, including RR-TB diagnosis, pre-treatment and on-treatment attrition, also accounting for RR-TB retreatment outcomes in case of treatment failure or relapse over such an extended period. While previous research has addressed some of these elements individually,^6^ none has integrated all components into a single comprehensive assessment.

To comprehensively evaluate Niger’s RR-TB programme’s performance and interpret its contribution to control, we evaluated the outcomes of all patients diagnosed with RR-TB over 15 years, from 2008 to 2022. We analysed pre-treatment attrition, time-to-treatment, on-treatment attrition, and whether all patients were offered a therapeutic solution, regardless of the complexity of the resistance pattern and number of RR-TB treatment episodes.

## METHODS

We conducted a retrospective observational cohort study, encompassing all patients diagnosed with RR-TB in Niger between 2008 and 2022.

### Setting and organisation of care

Niger, a resource-limited country, faces challenges such as political instability, insecurity, and a health care system requiring qualified human resources. RR-TB diagnostic tools evolved over three periods. From 2008 to 2013, RR-TB diagnosis relied on culture with phenotypic drug susceptibility testing (pDST). Between 2014 and 2018, Xpert testing was introduced, initially available in only four regions. From 2019 onwards, the Xpert network expanded significantly, reaching 71 machines nationwide. Since 2022, Xpert has been the initial TB diagnostic tool. The National TB Reference Laboratory (NRL), which performs culture, pDST, second-line probe assays (SL-LPA), and Xpert MTB/XDR, managed the Xpert sites. These sites were linked to RR-TB treatment units to ensure linkage to care. The Institute of Tropical Medicine laboratory provided culture and complete molecular and pDST for enrolled patients, and in case of failure or relapse. Patients received free RR-TB treatment at four units (Niamey, Maradi, Zinder, and Tahoua). Patient care included clinical evaluations, biological and bacteriological assessments, chest X-rays, and electrocardiograms adapted to their treatment regimen. Box 1 details the treatment regimens used. We provided social support, including food packages, transport stipends, and free management of adverse events. Staff dispensed treatment under directly observed therapy, except on weekends and public holidays, when patients self-administered their medication. Home visits were conducted for patients facing adherence challenges. Treatment outcomes followed WHO definitions, apart from ‘definitive treatment success’, which also considered outcomes after retreatment for failure or relapse.

Box 1. Treatment regimens for the first RR-TB episode for patients treated from 2008 to 2022 in Niger.

**SLI/FQ-STR:** used between 2008 and 2021, consisted of an FQ (gatifloxacin [G] or moxifloxacin [Mfx]), SLI (kanamycin [K] or amikacin [Am]), prothionamide (Pto), clofazimine (Cfz), high-dose isoniazid (Hh), pyrazinamide (Z), and ethambutol (E).

**Modified STR:** same as SLI/FQ-STR, but with replacement of K or Am by linezolid (Lzd) in case of initial hearing loss or hearing loss > 20 dB that appeared during treatment to prevent severe ototoxicity requiring hearing aids.

**BDQ/FQ-STR:** an all-oral regimen, has replaced SLI/FQ-STR as the first RR-TB treatment since 2021 and includes BDQ, high-dose levofloxacin (Lfx), linezolid (Lzd), clofazimine (Cfz), high-dose isoniazid (Hh), prothionamide (Pto), and pyrazinamide (Z).

**Pre-XDR-TB STR:** implemented in 2021, this includes delamanid (Dlm), BDQ, linezolid (Lzd), clofazimine (Cfz), high-dose isoniazid (Hh), and pyrazinamide (Z) for patients diagnosed with pre-XDR-TB. Dlm was used, as pretomanid (Pa) was unavailable in Niger until April 2025.

**Individualised regimen:** used to treat fewer patients with contraindications to the above-mentioned regimens due to comorbidities.

**RR-TB retreatment, RR-TB retreatment:** in patients in need of RR-TB retreatment (due to failure or relapse after initial treatment) after SLI/FQ-STR or modified STR, we used individualised BDQ-based treatment regimens. For RR-TB retreatment after BDQ/FQ-STR, we recycled levofloxacin (Lfx), bedaquiline (BDQ), and linezolid (Lzd) and added amikacin (Am), delamanid (Dlm), and imipenem–cilastatin (Imp-Cln) as no other effective options remained.

### Data collection, management, and analysis

Data sources included Xpert site registers, NRL TB registers, patient cards, and RR-TB unit registers. All data were compiled into a national RR-TB database, using a unique serial number for each patient. We used R Studio software for data analysis and summarised continuous variables with median and quartiles, and categorical variables with proportions. We used survival analysis to evaluate predictors of time-to-on-treatment attrition (defined as death or LTFU). Follow-up time began on the date of first RR-TB treatment initiation. Patients who achieved a definitive successful outcome were censored. One patient who had two treatment failures but remained in care was censored at the study end, with no definitive outcome. In case of attrition (death, LTFU), the patient was considered to have experienced the event on the corresponding date. To construct a multivariable Cox regression model, we first conducted bivariate analyses. Predictors with *P* < 0.2 were included in the multivariable model. The final model included regimen and variables significantly (*P* value < 0.05) associated with attrition.

### Ethical statement

This study received approval from the Institutional Review Board of the Institute of Tropical Medicine, Antwerp, Belgium (reference number 18483669).

## RESULTS

Between 2008 and 2022, 872 RR-TB patients were diagnosed, of whom 725 initiated treatments. The annual average of patients starting RR-TB treatment rose from 22 between 2008 and 2013 (pDST) to 50 in 2014–2018 (regional Xpert testing) and 85 between 2019 and 2022 (decentralised Xpert testing). Throughout the 15-year study period, we showed a high treatment uptake, with 83.1% of diagnosed RR-TB patients starting treatment (Figure).

**Figure.**
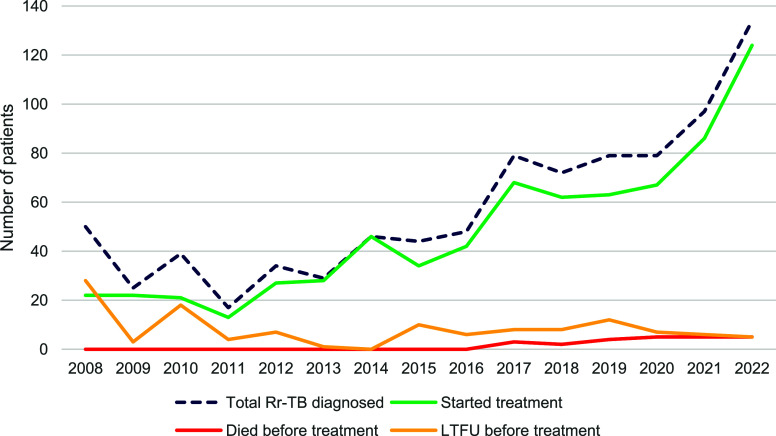
Trend of RR-TB treatment uptake, death, and loss to follow-up before treatment (2008–2022), Niger (*N* = 872).

The median time from RR-TB diagnosis to treatment initiation decreased significantly across periods: from 260 days (interquartile range [IQR]: 157–288) in the pDST period to 17 days (IQR: 12–18) with regional Xpert testing and 11 days (IQR: 6–15) with decentralised Xpert testing (*P* value = 0.004). Since the introduction of Xpert, there has been an increase in both the absolute number of diagnosed RR-TB patients and the number initiated on treatment. The absolute number of RR-TB patients achieving definitive treatment success rose more than four fold (an average of 20 per year for those starting treatment between 2008 and 2013 to 85 per year for the 2019–2022 cohorts). Pre-treatment attrition declined markedly from 31.4% during 2008–2013 to 12.8% in 2014–2018 and 12.6% in 2019–2022, whereas on-treatment attrition increased from 8.8% over 13.5%–19.8% in the above-mentioned periods, respectively (Table 1).

**Table 1.** Pre-treatment and on-treatment attrition of RR-TB patients in Niger from 2008 to 2022.

	Total	pDST (2008–2013)	Xpert in four states (2014–2018)	Xpert in all states (2019–2022)
	*N* (%)	*N* (%)	*N* (%)	*N* (%)
Total RR-TB diagnosed	872 (NA)	194 (NA)	289 (NA)	389 (NA)
Pre-treatment attrition	147 (16.9)	61 (31.4)	37 (12.8)	49 (12.6)
Started treatment	725 (NA)	133 (NA)	252 (NA)	340 (NA)
On-treatment attrition	133 (15.2)	17 (8.8)	39 (13.5)	77 (19.8)
Definitive treatment success^**A**^	592 (67.9)	116 (59.8)	213 (73.7)	263 (67.6)

NA = not applicable; LTFU = loss to follow-up; pDST = phenotypic drug susceptibility testing; RR-TB = rifampicin-resistant TB.

A One patient was excluded because the patient does not yet have a definitive outcome.

Table 2 summarises patient characteristics at the start of RR-TB treatment. At initiation, median patient age was 33 years (IQR: 26–42) and median body mass index (BMI) was 17.3 kg/m^2^ (IQR: 15.5–19.2); two thirds were underweighted. Fewer than 20% (*N* = 126, 17.4%) were female. HIV co-infection was present in less than 5%, and over 70% had high bacillary load. Previously treated for rifampicin-susceptible TB (RS-TB) were nearly 90%.

**Table 2.** Patient characteristics at admission and predictors associated with on-treatment attrition in Niger from 2008 to 2022 (*N* = 725).

	Total *N* = 725 (%)	Attrition *N* (%)	HR (95% CI)	aHR (95% CI)
Gender				
Men	599 (82.6)	101 (16.9)	0.60 (0.40–0.90)	0.56 (0.37–0.85)
Women	126 (17.4)	32 (25.4)	Ref	Ref
Age (years)				
<20	50 (6.9)	5 (10.0)	0.66 (0.27–1.64)	0.57 (0.23–1.42)
20–39	473 (65.2)	69 (14.6)	Ref	Ref
40–60	171 (23.6)	41 (24.0)	1.79 (1.21–2.63)	1.55 (1.04–2.32)
>60	31 (4.3)	18 (58.1)	5.70 (3.39–9.59)	7.24 (4.18–12.55)
HIV status^**A**^				
Negative	684 (95.3)	118 (17.3)	Ref	Ref
Positive	34 (4.7)	14 (41.2)	2.74 (1.57–4.76)	3.43 (1.90–6.18)
BMI (kg/m^2^)^**B**^				
<18.5	486 (67.5)	99 (20.4)	1.67 (1.09–2.55)	1.98 (1.26–3.09)
18.5–24.9	215 (29.9)	27 (12.6)	Ref	Ref
≥25	19 (2.6)	3 (15.8)	1.28 (0.39–4.22)	0.71 (0.21–2.40)
Initial bacillary load (SSM)^**C**^				
Low^**D**^	193 (26.7)	25 (13.0)	Ref	Ref
High^**E**^	530 (73.3)	108 (20.4)	1.61 (1.04–2.48)	1.71 (1.09–2.68)
Treatment regimen				
SLI/FQ-STR	477 (65.8)	81 (17.0)	Ref	
Modified STR	128 (17.6)	23 (18.0)	1.08 (0.68–1.71)	
All-oral	106 (14.6)	26 (24.5)	1.53 (0.98–2.38)	
Pre-XDR	9 (1.2)	3 (33.3)	1.90 (0.60–6.03)	
Individualised	6 (0.8)	0 (0.0)	<0.001 (NA)	
Period of treatment				
2008–2013	133 (18.3)	17 (12.8)	Ref	Ref
2014–2018	252 (34.8)	39 (15.5)	1.43 (0.79–254)	1.59 (0.86–2.95)
2019–2022	340 (46.9)	77 (22.6)	2.16 (1.27–3.67)	2.30 (1.29–4.09)

BMI = body mass index; SLI/FQ-STR = second-line injectable/fluoroquinolone-short treatment regimen; modified STR = modified short treatment regimen; pre-XDR = pre-extensively drug-resistant TB; SSM = sputum smear microscopy; HR = hazard ratio; Ref = reference category; aHR = adjusted hazard ratio; CI = confidence interval.

A Data missing for seven patients.

B Data missing for five patients.

C Data missing for two patients.

D Low initial bacillary load: initial sputum smear microscopy < 2+ grade.

E High bacillary load: initial sputum smear microscopy ≥ 2+ grade.

As a first RR-TB treatment, the SLI/FQ-STR demonstrated 79.2% sustained success (relapse-free success), 11.5% mortality, 4.2% LTFU, and 3.6% failure. The modified STR had similar (81.2%) sustained success, 16.4% mortality, 1.6% LTFU, and 0.8% failure. The all-oral BDQ/FQ-STR had the lowest (74.5%) sustained success, 16% mortality, 5.7% LTFU, and 3.8% failure. Differences in sustained success between SLI/FQ-STR and BDQ/FQ-STR were not significant (*P* = 0.3). Among four patients with treatment failure after BDQ/FQ-STR, one died before retreatment, one was LTFU, and two received individualised regimens based on the MTB strain resistance profile. One of those two patients experienced treatment failure again, and the other died 7 months later. Patients in need of RR-TB retreatment experienced high pre-retreatment mortality (18.8%). Among those treated, the sustained success rate was 87.1%. In patients treated for RR-TB, the definitive treatment success was 81.7%. One patient remained in care because of retreatment failure (Table 3).

**Table 3.** First treatment, retreatment, and definitive outcomes of RR-TB patients in Niger, 2008–2022.

	Cured/completed	Death	LTFU	Failure	Relapse
	*N* (%)	*N* (%)	*N* (%)	*N* (%)	*N* (%)
First RR-TB treatment					
First RR-TB treatment irrespective of regimen (*N* = 725)	572 (78.9)	95 (13.1)	28 (3.9)	22 (3.0)	8 (1.1)
First RR-TB treatment with SLI/FQ-STR (*N* = 477)	378 (79.2)	55 (11.5)	20 (4.2)	17 (3.6)	7 (1.5)
First RR-TB treatment with modified STR (*N* = 128)	104 (81.2)	21 (16.4)	2 (1.6)	1 (0.8)	0 (0.0)
First RR-TB treatment with all-oral STR (*N* = 106)	79 (74.5)	17 (16.0)	6 (5.7)	4 (3.8)	0 (0.0)
First RR-TB treatment with pre-XDR STR (*N* = 9)	6 (66.7)	2 (22.2)	0 (0.0)	0 (0.0)	1 (11.1)
First RR-TB treatment individualised regimen (*N* = 5)	5 (100.0)	0 (0.0)	0 (0.0)	0 (0.0)	0 (0.0)
RR-TB retreatment^**A**^					
Retreatment RR-TB individualised regimen (*N* = 23)	20 (87.1)	1 (4.3)	1 (4.3)	1 (4.3)	0 (0.0)
Definitive outcomes					
Definitive outcomes of RR-TB patients (*N* = 725)	592 (81.7)	102 (14.1)	30 (4.1)	1 (0.1)	NA

LTFU = loss to follow-up; SLI/FQ-STR = second-line injectable/fluoroquinolone-short treatment regimen; modified STR = modified short treatment regimen; pre-XDR = pre-extensively drug-resistant TB; RR-TB = rifampicin-resistant TB.

A Attrition before retreatment: 9, 6 due to death and 3 due to LTFU before starting retreatment.

In the multivariable model for definitive treatment success versus on-treatment attrition, men had a lower risk of attrition than women (adjusted hazard ratio [aHR]: 0.56; 95% confidence interval [CI]: 0.37–0.85). A higher risk of attrition was shown in patients with a low BMI (aHR: 1.98; 95% CI: 1.26–3.09), patients aged over 60 years (aHR: 7.24; 95% CI: 4.18–12.55), RR-TB-HIV co-infected patients (aHR: 3.43; 95% CI: 1.90–6.18), patients with a high baseline bacillary load (aHR: 1.71; 95% CI: 1.09–2.68), and those treated between 2019 and 2022 (aHR: 2.30; 95% CI: 1.29–4.09). Using an all-oral BDQ/FQ-STR was not significantly associated with attrition (hazard ratio: 1.53, 95% CI: 0.98–2.38; Table 2).

## DISCUSSION

This study evaluated 15 years of RR-TB management in Niger, showing a comprehensive view of RR-TB care and control, including diagnosis, pre-treatment, and on-treatment outcomes, as well as outcomes for those who underwent RR-TB retreatment. The roll-out of Xpert testing increased access to RR-TB diagnosis and shortened time-to-treatment initiation, thus improving treatment coverage. Moreover, treatment success was high for first and retreatment RR-TB episodes. Over time, the absolute number of patients achieving definitive treatment success increased more than four fold, likely contributing to RR-TB control and a declining incidence. Specifically, the incidence rate dropped from 3.1 to 2.5 per 100,000 between 2015 and 2020, approaching the End TB Strategy’s 20% reduction target.^1^

The implementation and scale-up of the Xpert network in Niger greatly enhanced RR-TB diagnosis and accelerated treatment initiation. These findings are consistent with a systematic review from sub-Saharan Africa, which confirmed Xpert’s role in improving diagnosis and facilitating timely treatment.^7^ Despite a 16.9% pre-treatment attrition rate, Niger achieved 83.1% treatment uptake throughout the study period, comparable to the 18% reported in a review by Jouego et al.^8^ Although the absolute number of diagnosed patients increased substantially, pre-treatment attrition reduced over time, indicating improved access to care in Niger.

First RR-TB treatment outcomes were good, with 78.9% treatment success, much higher than 50%–65% global success reported in the same period.^1^ This success can probably be attributed to the ‘cascade of treatment regimen’ approach, relying on a sequence of standardised regimens.^5^ The cascade approach included a rifampicin-based first-line regimen for RS-TB, a second-line regimen (fluoroquinolone, second-line injectable, and other drugs) for RR-TB patients with strains susceptible to fluoroquinolones, and a third-line regimen (BDQ plus other drugs) for RR-TB treatment failure or relapse, or pre-XDR-TB. This approach ensured patients consistently had access to effective regimens with little or no transmission of highly resistant, hard-to-treat TB. However, one patient experienced definitive treatment failure after receiving the WHO-recommended all-oral BDQ/FQ-STR regimen introduced in 2021 and even after a subsequent individualised retreatment regimen. This patient remained without treatment options, which led to Niger’s first confirmed extensively drug-resistant TB (XDR-TB, TB resistant to fluoroquinolones and BDQ) case. The emergence of BDQ resistance is increasingly concerning. In Mozambique, 14% of rifampicin-resistant *Mycobacterium tuberculosis* (MTB) strains were also resistant to BDQ,^9^ highlighting the urgent need for vigilant resistance surveillance.

While pre-treatment attrition improved over time, on-treatment attrition worsened. Predictors of on-treatment attrition included female gender, older age, HIV co-infection, low BMI, a high initial bacillary load, and treatment starting between 2019 and 2022. High baseline bacillary load,^10,11^ HIV co-infection,^12–15^ older age,^14,16–18^ and underweight^14,19^ are known predictors of attrition. While many studies linked men to higher attrition risk, our findings revealed greater vulnerability among female patients in Niger.^16,20,21^ We speculate that this may be attributed to the socio-economic realities in Niger, where women face financial dependence on men, require male permission to access health care, and have limited autonomy. These constraints may delay care-seeking, resulting in late presentation and higher early mortality. Additionally, patients treated between 2019 and 2022 had higher attrition rates. Xpert test introduction has substantially reduced the delay in initiating treatment and increased the number of patients receiving care. Conversely, it resulted in higher on-treatment attrition in 2019–2022. This phenomenon can be attributed to severely ill patients, who, despite being promptly treated, may succumb to treatment early, thus contributing to the observed high attrition. Mollel et al.^22^ reported a similar finding in Tanzania. Furthermore, the COVID-19 outbreak may have contributed, as co-infection may have increased mortality, while access to routine care worsened, thus increasing LTFU.

To mitigate on-treatment attrition, Niger’s National Tuberculosis Programme and its partners should focus on early TB and RR-TB detection using chest radiography with computer-aided detection, more intensive monitoring, and care for vulnerable populations. Addressing modifiable factors like low BMI by reinforcing nutritional support for severely malnourished patients and strengthening care for HIV-co-infected patients and the elderly, and adopting a multidisciplinary approach and free management of comorbidities, is essential. Improving women’s TB care access through targeted, gender-sensitive strategies may further reduce attrition.

This study has several strengths, primarily due to its comprehensive 15-year assessment of RR-TB control in Niger, offering a countrywide view on diagnosis, treatment uptake, and outcomes, including retreatment. This large timeframe and national scale underscore the study’s uniqueness. The study relied on a robust national RR-TB database, integrating both laboratory and clinical data. Continuous data verification ensured completeness and reliability. Missingness was minimal (two missing values for bacillary load, five for HIV status, and seven for BMI), which probably did not affect overall findings.

This study has some limitations. The retrospective cohort design was a first limitation, as patients were not randomly assigned to treatment regimens; hence, causality could not be assessed. Improved RR-TB diagnosis and timely treatment over time may have introduced selection bias. Patients who died or LTFU before treatment could skew on-treatment mortality and LTFU rates, thus affecting the attrition analysis. Moreover, pre-treatment attrition predictors could not be explored, as individual-level data were only available for those who initiated treatment. Finally, unlike in previous studies,^4,5,23^ we did not report adverse events, a limitation, as these affect both adherence and attrition.

## CONCLUSION

In Niger, between 2008 and 2022, Xpert MTB/RIF roll-out drastically reduced diagnostic delays and pre-treatment attrition. RR-TB treatment uptake was 83.1% among those diagnosed, and 81.7% of treated patients achieved definitive success, well above global averages (50%–65% in the same period). Niger’s strong performance in a challenging context likely advanced RR-TB control and offers a replicable model for similar settings. However, on-treatment attrition associated with female gender, older age, low BMI, high baseline bacillary load, RR-TB/HIV co-infection, and recent treatment initiation remains a concern.
